# The Quit Together couples-focused pilot randomized trial of tobacco cessation for pregnant smokers

**DOI:** 10.1016/j.ejogrb.2025.03.010

**Published:** 2025-03-07

**Authors:** Cristian I. Meghea, Marina D. Dascal, Rana Jaber, Alexandra Brinzaniuc, Alexandra Onisor, Razvan M. Chereches, Dan Mihu, Cristian I. Iuhas, Florin Stamatian, Daniel Muresan, Gabriela Caracostea, Kristie Foley, Adriana Baban, Thomas C. Voice, Ken Resnicow, David W. Wetter, Oana M. Blaga

**Affiliations:** a Department of Obstetrics, Gynecology and Reproductive Biology, College of Human Medicine, Michigan State University, MI, USA; b Center for Health Policy and Public Health, College of Political, Administrative and Communication Sciences, Babeș-Bolyai University, Cluj-Napoca, Romania; c Department of Public Health, College of Political, Administrative and Communication Sciences, Babeș-Bolyai University, Cluj-Napoca, Romania; d School of Health Sciences, Northern Illinois University, IL, USA; e Iuliu Hatieganu University of Medicine and Pharmacy, Department of Obstetrics and Gynaecology Dominic Stanca Clinic, Cluj-Napoca, Romania; f Iuliu Hatieganu University of Medicine and Pharmacy, Department of Obstetrics and Gynaecology Clinic I, Cluj-Napoca, Romania; g Department of Implementation Science, Division of Public Health Sciences, Wake Forest University School of Medicine, NC, USA; h Department of Psychology, Babeș-Bolyai University, Cluj-Napoca, Romania; i Department of Civil and Environmental Engineering, College of Engineering, Michigan State University, MI, USA; j Department of Health Behavior and Health Education, School of Public Health, and Rogel Cancer Center, University of Michigan, USA; k Center for Health Outcomes and Population Equity, Department of Population Health Sciences and Huntsman Cancer Institute, University of Utah, Salt Lake City, UT, USA

**Keywords:** Motivation and problem solving, Dyadic efficacy, Pregnancy smoking, Couple intervention, Clinical trial

## Abstract

**Aims:**

This study reports on the feasibility and preliminary efficacy of prenatal and postnatal couples-focused telephone counseling for pregnant tobacco smokers.

**Design and setting:**

This pilot randomized controlled trial (RCT) was conducted online in Romania and enrolled a total of 90 pregnant smokers and 77 of their life partners.

**Participants:**

90 adult pregnant smokers and 77 of their life partners were randomized either to (1) Motivation and Problem Solving (MAPS) telephone counseling (n = 45 pregnant smokers who received up to 8 pre/postnatal telephone counseling sessions and n = 40 partners who received up to 4 sessions) or (2) usual care (n = 45 pregnant smokers and n = 37 partners).

**Measurements:**

The primary outcomes were maternal 7-day self-reported and biochemically verified tobacco point-prevalence abstinence (PPA) and prolonged abstinence (PA) between birth and three months postpartum. Unadjusted group comparisons were used to assess preliminary intervention efficacy.

**Findings:**

20 % of women in the intervention group reported PPA, compared to 15.6 % (p = 0.58) in the usual care group. Women (n = 15) who received three or more counseling sessions had higher PPA (40.0 vs 15.6 %, p *<* 0.05) than women in the usual care group. 8.9 % of the women in the intervention group had biochemically verified abstinence compared to 4.4 % (p = 0.67) in the usual care group, with a significantly higher rate among women who received at least three counseling sessions (26.7 % vs 4.4 %, p = 0.03).

**Conclusion:**

A prenatal and postnatal couple-focused telephone counseling intervention for pregnant smokers is feasible and provides preliminary efficacy of cessation and postpartum abstinence when a minimum intervention dosage is delivered.

## Introduction

Tobacco smoking during pregnancy is harmful to the mother and fetus, including fetal growth restriction, preterm birth, sudden infant death syndrome, placental abruption, and childhood behavioral disorders [1]. Although most women understand the harmful effects of pregnancy smoking [2] and that quitting reduces risks [3], only half of women spontaneously quit when they find out they’re pregnant [4], and most others cannot quit on their own [5].

There is a current disagreement and ambiguity about the effects of the use of nicotine replacement therapy on the outcomes of pregnant women, despite their efficacy in the general population (Taylor et al., 2021). As a result, the options to help pregnant smokers quit are limited. Psychosocial interventions, including counseling, effectively increase pregnancy tobacco cessation [6]. These interventions, however, have largely been tested in high-income countries [6]. While the number of women smoking during pregnancy is decreasing in high-income countries, rates are increasing in low-to-middle-income countries (LMIC) [6].

Partner support for cessation may increase pregnancy quit rates and postpartum abstinence [7]. The few interventions that have included partner support were, in general, not successful in reducing pregnancy smoking and postpartum relapse [7–9]. The objective of this pilot randomized controlled trial was to evaluate the feasibility and preliminary efficacy of couples-focused telephone counseling intervention for tobacco smoking cessation and abstinence. The Quit Together couple intervention, including partner support and a focus on dyadic efficacy for smoking cessation [14], was a partnership between a US university and a Romanian university, obstetrics and gynecology hospitals and clinics in Romania, and other community partners [10].

## Methods

### Study design

The Quit Together project was a pilot randomized controlled trial of 90 Romanian pregnant smokers and 77 of their partners (registration date 31/07/2015, https://clinicaltrials.gov/ct2/show/NCT02512913). The sample size was a function of the enrollment criteria and the pilot nature of the trial. Participation was voluntary, all participants provided written informed consent, and the Michigan State University Institutional Review Board approved the study. The protocol of the trial was published elsewhere [10].

### Participants

To be eligible, women had to be 18 or older, pregnant, a current smoker of traditional cigarettes (even if occasionally), living with a partner, and willing to provide the partner’s contact details. Partners had to be 18 or older. Women whose partners declined participation after repeated offers to enroll in the study were still included. The participant enrollment trial was guided by the sample size in the project proposal and by practical considerations, including budgetary and time constraints.

### Recruitment and randomization

Recruitment took place online from June 2017 to May 2019 via a project website where women were directed mostly through online ads and posts but also medical professionals, perinatal educators, ads in hospitals, or other sources. The website hosted a link to a Qualtrics [11] form with a self-administered screening survey, consent form, and the baseline survey. We used block randomization [12] and allocated participants to the Motivation and Problem (MAPS) intervention group (n = 45 women and 40 partners) or the usual care (UC) group (n = 45 women and 37 partners). Allocation was concealed participants and investigators.

### Interventions

The intervention group received telephone cessation counseling sessions during pregnancy and 1-month postpartum. The number and the frequency of counseling sessions were determined individually [13–15]. Although there was no prescribed dosage of sessions, the counselors offered generally up to 6 prenatal sessions for women and up to 3 for partners and up to 2 postnatal sessions for women and up to 1 for partners. The counselors were certified psychotherapists who underwent multiple written and face-to-face training sessions with Motivational Interviewing specialists on the Motivation and Problem Solving (MAPS) framework that considers the social and life-related aspects of substance use in addition to tobacco cessation [13–17]. MAPS combines motivational strategies and problem-solving skills to enhance smoking cessation among women and to encourage partner support by enhancing the couples’ dyadic efficacy to quit smoking. The counseling sessions were audio recorded with the participants’ consent to ensure that the counselors were delivering the intervention properly.

The intervention development and cultural adaptation, the content for each counseling session, and the interventionists’ training are detailed elsewhere [10]. The women in the UC group received a one-page leaflet describing the importance of smoking cessation for the mother and the baby and a list of strategies the partner could use to help mothers quit smoking.

### Retention

Each participant was contacted on three consecutive days at varying times to maximize retention during the delivery of counseling sessions and follow-up phase. Women received US$10 (50 RON) gift cards after recruitment and one after birth, and partners received one US$10 (50 RON) gift card at recruitment.

### Follow-up

Participants were asked to complete a questionnaire three months postpartum. Women who self-reported as quitting smoking were offered a saliva test kit sent to their homes. The test was to be self-administered with guidance from a research team member over the phone. Women who self-reported ex-smokers but declined the saliva test were coded as smokers.

### Measures

Dyadic efficacy for smoking cessation [9] was evaluated using the average score of an 8-item instrument designed to assess the women’s confidence that they can work with their partners to quit and stay smoke-free, rated on a scale from 0 (not at all confident) to 100 (extremely confident). An adapted, 5-item version of the Bodenmann Dyadic Coping tool [9,18] assessed how the dyad coped with the stress related to quitting by rating statements (i.e., “my partner and I engage in serious discussions about smoking-related problems and think through what has to be done”) from 1 (very rarely) to 6 (very often). Importance, confidence, and readiness to quit and stay quit (for the follow-up) were assessed using one question each, with answers from 1 (low) to 10 (high). Self-efficacy was measured using the Romanian version of the General Self Efficacy scale [19], where 10 items are scored between 1 (not at all true) and 4 (exactly true), total score ranging from 10 to 40, with a higher score indicating greater self-efficacy level. The four-item (PHQ-4) patient health questionnaire [20] was used to evaluate the extent to which women experience anxiety and depression symptoms. Women were also asked to rate their partners’ support in their smoking cessation attempts using three positive (items 1, 7, and 9) and three negative (items 2, 3, and 7) items from the Partner Interaction Questionnaire [21]. Nicotine dependence at baseline (Table 1) was assessed using the Heaviness of Smoking Index [22] (HSI), coded as “low dependence” if the HSI score was *<* 2 and “moderate-heavy nicotine dependence” otherwise. Participant satisfaction was used to assess the feasibility of the intervention using a tailored client satisfaction questionnaire. Another feasibility measure was the follow-up rate. Intervention participation (at least one counselling session) and counselling dosage (number of sessions) were also assessed.

The primary outcomes were the maternal 7-day self-reported point prevalence abstinence (PPA), point abstinence verified by salivary cotinine (*<*10 ng/mL), and self-report prolonged abstinence (PA) assessed on a binary scale three months after birth (*<*5 cigarettes since birth). Other self-reported outcomes included reduced smoking between enrollment and follow-up, coded binary (fewer vs. more or the same number of cigarettes compared to baseline), and the number of quit attempts during pregnancy.

### Statistical analysis

Data were summarized using counts and proportions for categorical variables and mean (M) and standard deviation (SD) for continuous variables. Intervention and UC groups at baseline and follow-up were compared using the chi-square test, t-tests, or Fisher exact tests, as appropriate. Intention-to-treat analyses were used for the primary outcomes, with pregnant smokers lost to follow up coded as smokers. The threshold for group differences in baseline variables to be considered for inclusion in multivariable outcome analyses was set at p *<* 0.10. The difference in outcome between the intervention and the UC group was considered significant if p *<* 0.05. All the analyses were conducted using Statistical Package for Social Sciences (SPSS) version 26.0.

## Results

**Fig. 1.**
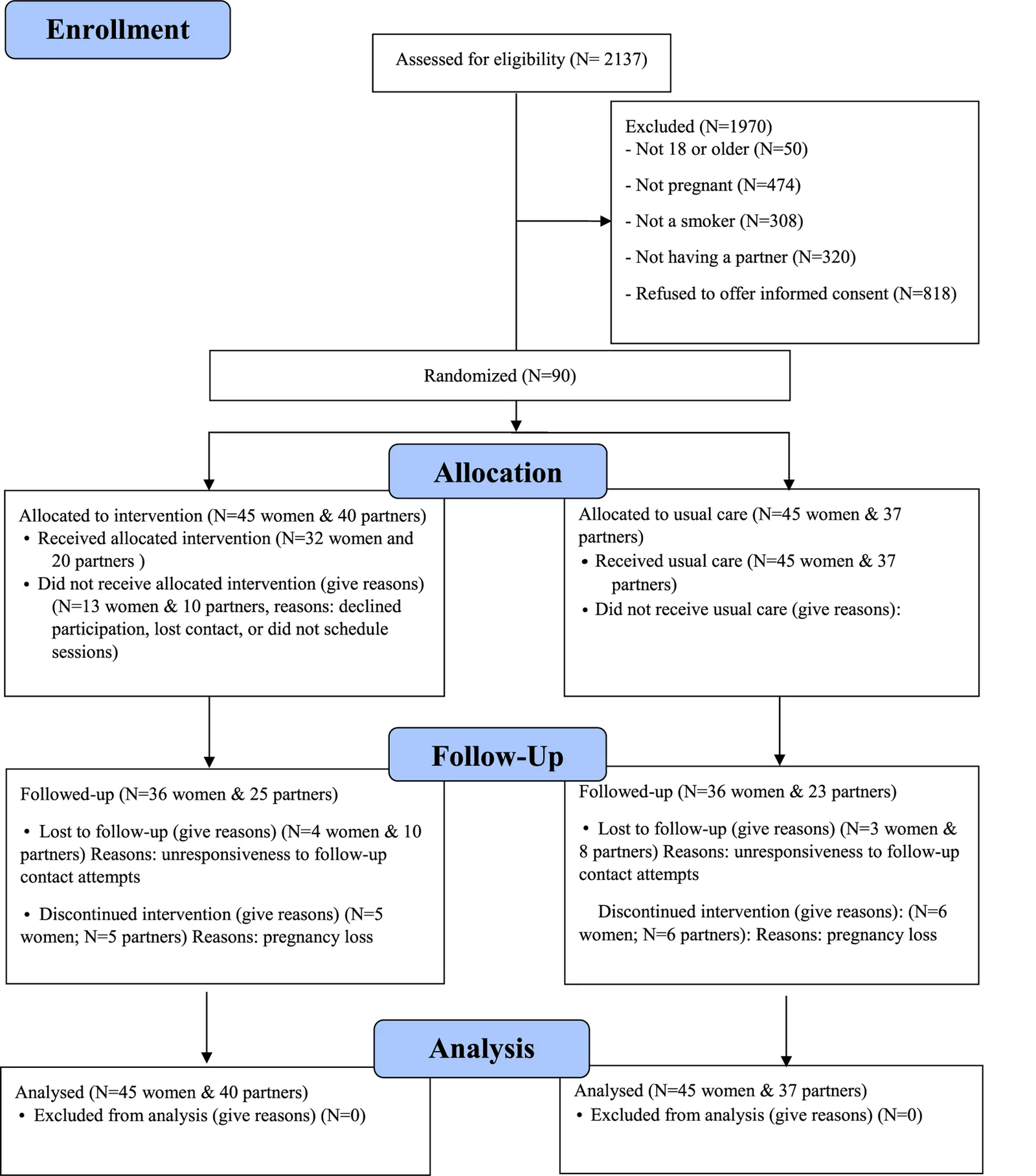
QT RCT participants CONSORT flowchart. *Note: these can be cumulative; one subject could fail to meet multiple inclusion criteria. ** Follow-up for women included a questionnaire and biochemical validation of smoking status through salivary cotinine assessment; it included a questionnaire for partners.

As shown in Fig. 1, n = 2,137 women completed the screening survey, of whom n = 985 were eligible, n = 167 consented, n = 131 filled out the baseline survey, and n = 90 were considered randomized intervention participants as they offered the contact details of their partners.

### Participants’ characteristics

Around 49 % of participants had a BA degree or higher, half had a monthly income below 3000 RON (~650 USD), 63 % were employed, 18 % had a chronic health condition, 73 % had a partner who smoked, and 65.6 % had moderate-severe nicotine dependence. There were no group differences in baseline characteristics of women (Table 1), so group comparisons were not adjusted for baseline covariates.

### Intervention feasibility and satisfaction

A total of 80 % (72/90; 36 intervention and 36 UC) of the pregnant smokers and 62 % (48/77; 25 intervention and 23 UC) of their partners were followed up at three months postpartum (Fig. 1). Participants were lost to follow up if they were unresponsive or had pregnancy loss. The follow-up rate was 91 % among the women who delivered a live baby, as 11 of the 18 women not followed up lost the pregnancy and the rest (n = 7) did not answer the phone. On a scale of 1 to 7 (very unsatisfied to very satisfied), pregnant smokers rated satisfaction with the counseling at 6.63 and 6.75 for counselors. Close to 97 % (30/31) said that would recommend the intervention to a friend (Table 2).

### Intervention participation and dosage

Approximately 71 % (32/45) of the women in the intervention group and 50 % (20/40) of their partners received at least one tobacco cessation counseling session (Table 3). The number of prenatal and postnatal counseling sessions ranged between 0 and 8 for the pregnant smokers (0–6 prenatal sessions and 0–3 postnatal sessions) and between 0 and 4 sessions (0–3 prenatal sessions and 0–2 postnatal sessions) for their life partners. Among women who received any counseling, 62.5 % (20/32) received at least two sessions, and 46.9 % (15/32) received at least three sessions. Among partners who received sessions, 47.8 % (11/23) received at least two sessions, and 30.4 % (7/23) received at least three sessions.

### Intervention outcomes

Twenty percent of the pregnant smokers in the intervention group reported 7-day point-prevalence smoking abstinence (PPA) at three months postpartum, compared to 15.6 % (p = 0.58) in the UC group, as reported in Table 4. Pregnant smokers who received three or more counseling sessions had a higher PPA at 3 months postpartum (40.0 vs. 15.6 %, p *<* 0.05), than UC women. 8.9 % % of the pregnant smokers in the intervention group had biochemically verified abstinence at 3 months postpartum versus 4.4 % (p = 0.67) in the UC group, with a significantly higher rate among women who received at least three counseling sessions (26.7 % vs. 4.4 %, p = 0.03). The rates of prolonged abstinence (PA) postpartum, measured as *<* 5 cigarettes smoked since birth, were similar in the intervention group and the UC group (15.6 % vs. 17.8 %, p = 0.78). A greater percentage woman among those who received 3 or more counseling sessions reported PA compared to the UC group (33.3 % vs 17.8 %, p = 0.21), although this difference was not statistically significant. As reported in Table 5, among life partners (all males) who smoked tobacco, more reported reduced smoking in the intervention group than the UC group (77.8 % vs. 35.7 %, p = 0.03).

## Discussion

To our knowledge, this was the first couples-focused pregnancy smoking cessation pilot trial implemented in a Central and Eastern European country and one of a few in low and middle-income countries. MAPS showed feasibility, was well-received by participants, and had high levels of reported satisfaction. Almost three quarters of the pregnant smokers participated in counseling sessions, with approximately half receiving at least three prenatal or postnatal sessions. A greater proportion of women in the intervention group reported seven-day point prevalence abstinence at three months postpartum, both self-reported and biochemically confirmed, with statistically significant effects among those who received at least three prenatal or postnatal counseling sessions. Those who received at least three prenatal or postnatal counseling sessions reported twice the rate of prolonged postpartum abstinence, defined as smoking ≤ 5 cigarettes in the first three months postpartum, compared to the UC group, although the difference was not statistically significant. The intervention increased the share of smoking life partners who self-reported reduced smoking.

This being a pilot trial, the small sample size may limit the generalizability of our findings. We intended to test the feasibility and initial efficacy of the intervention, with a planned fully powered trial for broader generalizability. It was challenging to enroll and engage life partners in the counseling. Although most of the life partners enrolled, only slightly over half received any counseling sessions. This may have limited the efficacy of the intervention through partner support, which was an intended mechanism.

Despite these limitations, the study findings are of interest because they inform future dyadic interventions. An additional strength is the fact that the intervention offered counseling sessions during pregnancy and early after birth, a period of time when many former smokers relapse. Compared to the other counseling interventions for pregnancy tobacco cessation [6], our intervention may yield higher cessation and continued postpartum abstinence rates when a minimum intervention dosage is delivered. A 2017 Cochrane review [6] reports an average 13 % cessation rate (11 % among those with biochemically validated cessation) among pregnant smokers who received counseling interventions, with a relative effect of RR =1.44 (RR =1.23 biochemically validated). The same review reports average RR = 1.59 on smoking abstinence at zero to five months postpartum. Our pilot intervention suggests higher self-reported 7-day abstinence rates at three months postpartum among those with 3 or more prenatal and postpartum counseling sessions (40 %, RR = 2.56) and higher verified postpartum point prevalence abstinence rate among those with 3 + sessions (26.7 %, RR = 6.06). The self-reported prolonged 3-month postpartum abstinence rate was also higher, at 33.3 % (RR = 1.87).

A 2012 systematic review of interventions for smoking cessation during pregnancy with partner support [7] found no RCTs that showed increased prenatal cessation or postnatal abstinence among maternal smokers. The study by McBride et al. (2004) is, to the best of our knowledge, the most similar to our couple-focused intervention. The study enrolled pregnant women who smoked or quit the 30 days before pregnancy and their partners. The intervention consisted of motivational interviewing based prenatal and postnatal (up to 4 months) counseling sessions to women and their partners. There were no intervention effects on postpartum abstinence rates compared to usual care.

## Conclusions

The findings of this pilot trial suggest that a couple-focused motivational and problem-solving intervention was feasible, showed high levels of participant satisfaction, and showed some evidence of efficacy during pregnancy and postpartum. The intervention may result in higher abstinence rates especially among pregnant smokers who receive at least 3 counselling sessions. The reduced smoking among partners could be a mechanism of intervention effect. A fully powered randomized RCT is needed to assess the effectiveness of a MAPS telephone counseling pregnancy couples-focused smoking cessation intervention.

## Figures and Tables

**Table 1:** Baseline characteristics.

Pregnant smokers	Usual Care (n = 45)		Intervention (n = 45)		
					
	n	%	n	%	p-value

Education					
< high school	6	13.30	3	6.70	
≥ high school < bachelor	18	40.00	19	42.20	0.714
Bachelor’s degree	14	31.10	17	37.80	
Graduate degree	7	15.60	6	13.30	
Monthly income (1 RON = $0.22)					
0–1500 RON	12	27.30	8	18.20	
1501–3000 RON	10	22.70	14	31.80	0.370
3001–5000 RON	13	29.50	17	38.60	
>5000 RON	9	20.50	5	11.40	
Employed	30	66.70	27	60.00	0.512
At least one chronic condition^a^	6	14.00	10	22.70	0.291
Moderate to severe psychological distress – vs low (PHQ-4)	36	83.70	40	90.90	0.313
Moderate to severe nicotine dependence – vs. low	33	73.3	26	57.8	0.120
Partner is smoking	24	70.60	25	75.80	0.633
First pregnancy	23	51.1	29	64.4	0.200
Wanted pregnancy	44	97.8	40	90.9	0.203
Have support to help with pregnancy and baby	43	95.6	41	91.1	0.677
Importance to quit smoking” (average score ± standard deviation)	9.0 ± 1.76		8.9 ± 1.66		0.712
Confidence in quitting smoking (average score ± standard deviation)	43 ± 59.0		42 ± 60.3		0.838
Dyadic efficacy score (average score ± standard deviation)	65.8 ± 21.8		67.1 ± 25.4		0.818
Dyadic coping score (average score ± standard deviation)	5.2 ± 1.1		4.8 ± 1.2		0.147
Partner Interaction questionnaire score (PIQ-) (average score ± standard deviation)	2.9 ± 1.2		3.1 ± 1.3		0.293
Partner Interaction questionnaire score plus (PIQ + ) (average score ± standard deviation)	4.1 ± 1.6		3.9 ± 1.3		0.513
Perceived general self-efficacy score (average score ± standard deviation)	32.3 ± 12.8		29.4 ± 11.1		0.255

Note: All percentages are of participants with valid, non-missing information

a Reporting at least one of the following (Asthma, diabetes, hypertension, thrombophilia, obesity, anemia, lupus, epilepsy, cancer, or any health condition).

**Table 2:** Satisfaction with the intervention among pregnant women (n = 31) and their life partners (n = 20).

Self-reported satisfaction scores from 1 (very unsatisfied) to 7 (very satisfied)	Pregnant smokers (Average ± SD)	Partners (Average ± SD)

In general, with the telephone counseling program	6.63 ± 0.66	6.45 ± 0.89
With how the counselors talked to you	6.75 ± 0.51	6.65 ± 0.75
With the way the telephone counseling sessions helped you in quitting or reducing smoking	6.00 ± 1.44	6.06 ± 1.39
With the way the counseling sessions fit your needs at that specific time	6.25 ± 1.27	6.20 ± 0.89
With the number of counseling sessions received	6.28 ± 1.09	6.05 ± 1.19
With the way the telephone counseling sessions helped you offer your partner support to quit	Not asked	6.25 ± 1.12
	Pregnant smokers n (%)	Partners n (%)
Would you recommend this tobacco smoking cessation program to a friend	30 (96.8)	19 (95.0)

**Table 3:** Counseling intervention dosage.

	n (%)

Pregnant smokers with 1 + sessions	32 (71.1)
Life partners with 1 + sessions	20 (50)
**Among those who received sessions**	
Pregnant smokers with 2 + sessions	20 (62.5)
Life partners with 2 + sessions	11 (47.8)
Pregnant smokers with 3 + sessions	15 (46.9)
Life partners with 3 + sessions	7 (30.4)

**Table 4:** Maternal postpartum point prevalence abstinence (PPA) and prolonged abstinence (PA).

Intention to treat analyses	Usual Care (n = 45) n (%)	Intervention (n = 45)n (%)	p-value

PPA: Seven-day point prevalence abstinence postpartum	7 (15.6)	9 (20.0)	0.58
Verified abstinence – saliva	2 (4.4)	4 (8.9)	0.68
PA: Smoked ≤ 5 cigarettes since birth	8 (17.8)	7 (15.6)	0.78
Among pregnant smokers with 3 + counseling sessions	Usual Care (n = 45) n (%)	Intervention (n = 15)n (%)	p-value
PPA: Seven-day point prevalence abstinence postpartum (3 + sessions)	7 (15.6)	6 (40)	<0.05
Verified abstinence – saliva (3 + sessions)	2 (4.4)	4(26.7)	0.03
PA: Smoked ≤ 5 cigarettes since birth (3 + sessions)	8 (17.8)	5 (33.3)	=0.21

**Table 5:** Quit attempts and reduced smoking at postpartum follow-up.

	Usual Care (n = 33) n (%)	Intervention (n = 32)n (%)	p-value

Quit attempts, pregnant smokers (mean ± SD)	2.9 ± 2.1	2.7 ± 2.4	0.82 ^a^
Smoked less vs. enrollment in the program (pregnant smokers)	27 (75.0)	26 (74.3)	0.95
Smoked less vs. enrollment in the program (life partners)	5 (35.7)	14 (77.8)	0.03

## Use of Abbreviations

QT: Quit Together
RCT: Randomized Controlled Trial
MAPS: Motivation and Problem Solving
PPA: Point-prevalence abstinence
PA: Prolonged abstinence
LMIC: Low-to-middle-income countries
PHQ-4: Patient Health Questionnaire
HSI: Heaviness of Smoking Index
PIQ: Partner Interaction Questionnaire
SD: Standard deviation
SPSS: Statistical Package for Social Sciences
RR: relative risk
UC: Usual Care
US: United States
